# Immunomodulatory Effects of Clozapine: More Than Just a Side Effect in Schizophrenia

**DOI:** 10.2174/1570159X22666231128101725

**Published:** 2024-11-28

**Authors:** Andrea Amerio, Luca Magnani, Gabriele Arduino, Fabio Fesce, Renato de Filippis, Alberto Parise, Alessandra Costanza, Khoa D. Nguyen, Daniele Saverino, Domenico De Berardis, Andrea Aguglia, Andrea Escelsior, Gianluca Serafini, Pasquale De Fazio, Mario Amore

**Affiliations:** 1 Department of Neuroscience, Rehabilitation, Ophthalmology, Genetics, Maternal and Child Health (DINOGMI), Section of Psychiatry, University of Genoa, Genoa, Italy;; 2 IRCCS Ospedale Policlinico San Martino, Genoa, Italy;; 3 Psychiatry Unit, Department of Health Sciences, University Magna Graecia of Catanzaro, Catanzaro, Italy;; 4 Department of Geriatric-Rehabilitation,, Azienda Ospedaliero-Universitaria di Parma, Parma, Italy;; 5 Department of Psychiatry, Faculty of Medicine, University of Geneva (UNIGE), Geneva, Switzerland;; 6 Department of Psychiatry, Faculty of Biomedical Sciences, University of Italian Switzerland (USI) Lugano, Switzerland;; 7 Department of Microbiology and Immunology, Stanford University, Palo Alto, CA, USA;; 8 Tranquis Therapeutics, Palo Alto, CA, USA;; 9 Department of Experimental Medicine (DiMeS), Section of Human Anatomy, University of Genoa, Genoa, Italy;; 10 NHS, Department of Mental Health, Psychiatric Service for Diagnosis and Treatment, Hospital “G. Mazzini”, Teramo, Italy

**Keywords:** Clozapine, immunomodulation, microglia, neuroinflammation, schizophrenia, SSDs

## Abstract

Recent evidence suggests a possible relationship between the immune system and schizophrenia spectrum disorders (SSDs), as neuroinflammation appears to play a role in major psychiatric conditions. Neuroinflammation is as a broad concept representing a physiological protective response to infection or injury, but in some cases, especially if chronic, it may represent an expression of maladaptive processes, potentially driving to clinical dysfunction and neurodegeneration. Several studies are concurrently highlighting the importance of microglia, the resident immune cells of the central nervous system, in a huge number of neurodegenerative diseases, including multiple sclerosis, Alzheimer’s and Parkinson’s diseases, as well as SSDs. A more fundamental phenomenon of maladaptive coupling of microglia may contribute to the genesis of dysfunctional brain inflammation involved in SSDs, from the onset of their neurophenomenological evolution. Clozapine and other antipsychotic drugs seem to express a provable immunomodulant effect and a more specific action on microglia, while neuroactive steroids and nonsteroidal anti-inflammatory drugs may reduce some SSDs symptoms in add-on therapy. Given these theoretical premises, this article aims to summarize and interpret the available scientific evidence about psychotropic and anti-inflammatory drugs that could express an immunomodulant activity on microglia.

## 1 INTRODUCTION

Schizophrenia (SCZ) and schizophrenia spectrum disorders (SSDs) have been recently described as neurodevelopmental disorders without a clear and singular etiology [1]. The complexity of SCZ causes probably involves very intricate gene-environment interactions which occur within a broader framework of individual vulnerability and differential susceptibility [2, 3]. Recent research indicates that many of the significant gene loci associated with SCZ risk are related to the immune system, with several located in the Major Histocompatibility Complex (MHC) region on chromosome 6 (6p21.3-22.1) [4-6].

Although the contribution of the immune system to the pathogenesis of SSDs is not yet clear, various findings suggest that it may play an important role [4, 7-12] and, more in general, the existence of an important and complex interconnection between psychiatric disorders and immunity [11]. Contrary to previous beliefs that the blood-brain barrier (BBB) and the “immunological privileged status” of the brain would prevent communication between the immune system and the central nervous system (CNS), research has shown that many immune molecules are active and expressed in the nervous system (NS) and *vice versa* [12].

Van Kesteren and colleagues conducted a recent meta-analysis on post-mortem studies of brain tissues from individuals with SCZ [13]. They discovered a noteworthy rise in microglia density in the brains of SCZ patients relative to healthy controls (HCs), particularly in the temporal cortex, speculating a possible involvement of microglia and neuroinflammation in SCZ pathogenesis. However, the composition of macroglia, including astrocytes and oligodendrocytes, did not differ significantly between SCZ patients and HCs.

The above-mentioned conflicting and heterogeneous results have been more recently addressed by Snijders and colleagues [14], who proposed a more subtle alteration in microglial phenotype (loss of mature microglial markers without immune activation) as a key molecular characteristic for potential microglial involvement in SCZ.

It is plausible that such a finer phenotypical alteration may be explained through the idea of an initial maladaptive coupling between early microglia and CNS environment.

These novel findings and related interpretations may help overcome the traditional, yet inaccurate, dichotomy between ‘activated’ and ‘resting’ microglia. Additionally, the vague and broad concept of ‘neuroinflammation’ may also be consequently better defined and more accurately used to support etiopathogenetic hypotheses regarding the development of various neuropsychiatric conditions.

Crosstalk between microglia and neuro-glial environment is now recognized as essential for maintaining brain homeostasis [15, 16]. The role of microglia extends far beyond its traditional function in immune surveillance. Indeed, microglial cells are also indispensable for brain wiring, cross-talking with neurons in a bi-directional communication (based on the exchange of signals *via*, for example, structural proteins, neurotransmitters, neuropeptides, growth factors and cytokines), for regulating synaptic pruning [17], and, crucially, for the network neural synchronization [18]. Taken together, all these observations highlight the multilevel importance of microglia not only in the brain physiological development and homeostasis but also in neurologic and neuropsychiatric illnesses linked to neuronal loss and dysfunction [19].

Importantly, recent experimental studies show that events occurring during early ontogenetic phases of an organism's development, such as maternal infections, can result in long-term alterations in microglia physiology and in an abnormal brain development in the offspring [20, 21]. An early priming effect, mediating by several factors [22], could promote the above-mentioned maladaptive coupling between microglia and CNS environment contributing to the development of diseases within a general double-hit or multi-hit model [23, 24].

Microglia express distinctive phenotypic signatures that are contingent on the specific region of the brain in which they reside: different phenotypes express specific molecular markers [25], and certain subtypes have been associated with higher levels of free radicals production and pro-inflammatory cytokines secretion due to their defective phagocytosis [26]. We suppose that an altered incorporation into the CNS reflected in some of these microglial phenotypes, could be at least partially responsible for dysfunctional neuroinflammatory changes that are eventually involved in SSDs pathogenic process.

The term ‘incorporation’ primarily refers to a dynamical state of functional coupling, mediated by various intercellular factors and pathways of intracellular components, defining the “dialogic” dimension between microglia and other neuroglial elements. Qualitatively different forms of dynamical coupling are indeed possible as, for instance:

1) Symbiotic coupling: exchanges of molecules and interaction during homeostasis and development;
2) Productive and reactive coupling: response during infection, tissue regeneration after injury; physiological and limited neuroinflammation;
3) Maladaptive coupling: aberrant activity that can cause and support excessive neuroinflammation/ degeneration, potentially linked to neuropsychiatric conditions. This situation could also be termed “dis-incorporation”.

Therefore, the incorporated condition encompasses all potential adaptive forms of coupling (type 1 and 2), through which microglia can express physiological morpho-functional characteristics, including adequate and limited phenomena of neuroinflammation. On the contrary, the maladaptive coupling could be considered critical component in the SCZ and other SSDs etiopathogenesis, referring, for instance, to the bridge concept of “ basal irritation”, which is also considered phenomenological terms [27].

Therefore, we would like to reiterate the crucial significance of considering the multiple level of microglia pathogenetic involvement and the relevance of possible specific microglia-targeted interventions, also relating to each SCZ progression phase. Conventional psychotropic and other known immune-active drugs may firstly mitigate the microglia-mediated excess of neuroinflammation in SSDs, but also, and more importantly, potentially promote a phenotypical shift of microglia towards cellular assets that are more capable of expressing forms of functional coupling with the CNS environment [28-30].

Given these theoretical premises, we conducted a narrative review of the available evidence from the literature, centred on the main pharmacological treatments that exhibit an immunomodulant activity on microglia, with a particular emphasis on their relevance to SSDs.

## 2 CLOZAPINE AND OTHER ANTIPSYCHOTIC DRUGS

Clozapine (CLZ) is a second-generation antipsychotic, highly effective and recommended for the management of treatment-resistant SCZ [31, 32]. An, uncertain body of evidence also suggests beneficial effects on mania, treatment-resistant major depressive disorder, and rapid cycling bipolar disorder (BD) [33]. However, due to its disturbing side effect profile, CLZ is not a first-choice treatment for SCZ and its use is limited to around 5% of patients [34].

The mechanism through which CLZ reduces psychotic symptoms was historically detected in its capacity to antagonize the serotonin receptors 2A and C (*i.e*., 5-HT2A and 5-HT2C) and the dopamine receptors (DRs) (*i.e.,* DRD1-DRD4, and especially DRD2), also acting on various other targets such as adrenergic, histaminergic, GABAergic, NMDA and muscarinic receptors.

However, a certain role may be also played by the immunomodulatory activity of CLZ. Indeed, being a low molecular weight compound [35], CLZ can easily cross the BBB spreading to different CNS structures [36]. Moreover, the ability of CLZ to modulate microglia function recently emerged [37]. Many recent works concerning CLZ immunomodulating effect have been conducted *in vitro* and/or using animal models (rodents) of human neuropathological conditions (*e.g.,* experimental autoimmune encephalitis - EAE) [38]. Ongoing results can be interpreted mostly in the context of specific experimental conditions, taking into account potential confounding factors related to the artificially induced dis-incorporation of cells in cultures. Moreover, the majority of the studies do not compare CLZ with other antipsychotic drugs.

*In vivo* EAE murine model - *i.e.,* a model typically used to study multiple sclerosis (MS) [39] - showed that CLZ has a beneficial immunomodulatory effect. For instance, Ceylan and colleagues [37] proved that CLZ modulates microglial activity regulating the inflammatory effect of free iron. The release of iron in the CNS is one of the dysfunctional mechanisms of chronic inflammation that lead to neurodegeneration in MS. In this study, authors simulated MS chronic inflammatory damage by treating human microglial cells line 3 (HMC3) with free iron. The overall effect of CLZ pre-treatment in iron-treated HMC3 was notable: CLZ- treated models showed a decrease in the release of the inflammatory cytokine IL-6 and an increase in microglial viability after oxidative stress. Additionally, CLZ was found to normalize the effects of different dosages of free iron on the phagocytosis of dead neurons.

*In vitro* and *in vivo* studies showed that CLZ treatment reduces the production of pro-inflammatory cytokines and chemokines, such as CCL2 and CCL5, in the CNS [40], as well as the expression of activation markers on CNS-resident cells [41, 42], leading to a decreased infiltration of myeloid and CD4 T cells [39, 42]. Moreover, in EAE murine models, the extent of demyelination was less pronounced in rodents treated with CLZ compared to untreated ones [37]. While, some immunomodulatory effects of the antipsychotics have been proven not to be exclusive of CLZ [43]. In fact, other atypical antipsychotics, such as risperidone, olanzapine and quetiapine seem to share some immunomodulant properties [44-51], but CLZ appears to exhibit the most intense immunomodulatory effects.

However, it is important to note that these studies were conducted *in vitro* and/or using animal models of human neuropathological conditions, and further research is needed to evaluate the clinical significance of these findings in human subjects.

With regard to the immunomodulant action of antipsychotics, in a recent meta-analytical work [52] of 12 studies conducted on subject affected by a first episode of psychosis (FEP) (*i.e.,* studies durations range = 4-24 weeks; studies samples range = 24 to 83 FEP subjects; n = 5 studies evaluated treatment with risperidone alone) a significantly decreased level of some plasma/blood serum proinflammatory (*i.e*., IL-1β, IL-6, IFN-γ) and anti-inflammatory (*i.e*., IL-4, IL-10) cytokines was highlighted, after the administration of the above mentioned psychotropic drugs. Interestingly, IL-1β, IL-4 and TNF-α levels in FEP subjects resulted higher than those expressed by HCs at the baseline and the difference was annulled by treatment. The significance of TNF-α reduction was lost only after the exclusion of studies including subjects with a history of prior treatment with antipsychotics. Such results have been interpreted in line with previous works and at least partially coherent with other inconsistent studies [53-58]. Notably, IL-1β (*i.e*., reported as decreased after antipsychotic treatment in most metanalytical works), IL-6, and TNF-α are macrophage-derived cytokines and their production is also inducible in microglial cells [59]. Moreover, levels of various cytokines have been associated with positive schizophrenia symptoms’ scores, the intensity of negative symptoms in onset phases of SCZ and in FEP [54, 60, 61], the deficit syndrome, the blunted affect, the alogia, and total negative symptoms in SCZ patients [62].

The results described above could be accounted for by the potential impact of antipsychotics on microglia. This microglial interpretation was based on previous experimental insights about the effect of antipsychotics and dopamine itself on microglia [44, 46, 63-66]. Regarding the inter-drug difference, Romeo and colleagues [54] noted that quetiapine administration scarcely influences cytokines levels, while treatment with CLZ resulted in a more intense anti-inflammatory effect, associated with increased level of serum TNF-R1, serum TNF-R2 and serum IL-2R, as well as a pro-inflammatory rise of IL-6 production. In any case, the underlying general mechanism is unlikely to be linked simplistically to DRs antagonism [66, 67].

An enhanced effect produced by CLZ on microglia may also be related to the drug's ability to potentially alter the expression of DRs on immune cells. At least five main types of DRs (*i.e*., D1-like family: DRD1 & DRD5; D2-like family: DRD2, DRD3 & DRD4) are usually present on brain-resident microglia and - in greater quantities - also on other immune cells (*i.e*., monocytes and macrophages) [40, 63, 68]. The interaction between dopamine and T cells can modify inflammatory cytokine secretion [69, 70] and the same molecule acting on monocytes and macrophages can shift their phenotypic expression, thus enhancing a more functional phagocytic capacity [71].

Dopamine itself can also directly modulate microglia function [65]. In particular, CLZ - while directly antagonizing DRD1 and DRD2 on neurons surface - significantly enhances DRD1 and DRD2 expression on myeloid cells [40]. DRD1 and DRD2 are low-affinity DRs: their activity has been suggested to modulate immunity *via* creating an anti-inflammatory state [72]. Since they are low-affinity receptors, their activity can only occur when there are high concentrations of dopamine present in the local area. If the dopaminergic flood classically related to psychotic productivity could also be considered as a sort of pseudo-adaptive attempt to endogenously modulate microglia activity and to restore a more functional coupling, then using CLZ might result in an upregulation of these low-affinity receptors, thus possibly preventing a) the need to maintain this enhanced dopaminergic tone over time, and b) the consequent shift of equilibrium in an allostatic sense. These speculations focus on the complex interactions between CLZ, dopamine and DRs, thus highlighting that the underlying mechanism is unlikely linked simplistically to DRs antagonism [67].

Again, concerning microglia and CLZ more specifically, CLZ *in vitro* inhibits Ca^++^/Calmodulin with subsequent suppression of Akt/NF-kB-mediated neuroinflammatory responses in TLR4-activated microglia [73, 74]. NF-κB plays a crucial role in the regulation of gene expression in microglia. At the same time, a possible involvement of Akt in SCZ has been proposed [75]. Furthermore, considering *in vivo* murine model, LPS-induced (*i.e.,* peritonea administered) MHC class II expression on microglia seems to be substantially abrogated in the presence of CLZ [74]. Moreover, CLZ dose-dependently attenuates clinical signs in chronic EAE, even if used late, in the advanced stages of the disorder, with positive effects on histological markers such as demyelination [42].

*In vitro*, CLZ modulates microglial activity by regulating the trigger effect of other soluble factors - *e.g*., the already mentioned free iron, iron overload is also a hallmark of CNS ageing and is associated with several neurodegenerative disorders [76]. In 2020, Giridharan *et al*. [77] investigated the production of IL-1α, IL-1β, IL-2, IL-4, IL-5, IL-6, IL-10, IL-17, IL-18, INF-γ, and TNF-α in the unstimulated and polyriboinosinic-polyribocytidilic acid (poly (I:C))-stimulated primary microglial cell cultures. The authors demonstrated that some antipsychotic drugs (*i.e.,* CLZ, risperidone, and haloperidol) influence the balance between pro- and anti-inflammatory cytokines upon cell stimulation by poly (I:C).

In addition, CLZ inhibits NLRP3 inflammasome activation, significantly reducing the expression of the proinflammatory cytokines, and its effect is comparable to CRID3, a well-known NLRP3 inflammasome inhibitor. Several studies have supported the poly (I:C) experimental paradigm as a very powerful neurodevelopmental animal model of SCZ and other relevant brain disease. Poly (I:C) was used to stimulate the rat primary mixed glial cell cultures enriched for microglia, to mimic the SCZ condition *in vitro.* Interestingly, it also appears to simulate a form of maternal immune activation (MIA) [78, 79]. MIA could be considered as a paradigmatic example of an early priming effect, and acting on microglia during the first phases of ontogenetic development. In the last study presented [77] the protective effect of CLZ on NLRP3 inflammasome activation has been further confirmed by measuring the mRNA levels: CLZ reduced the level of NLRP3 expression by 57%, which was higher than the reduction seen with the NLRP3 inflammasome inhibitor CRID3 (45%).

From *in vitro* and animal studies, although not specifically focused on human SCZ, a plausible and complex immunomodulatory effect (also concerning microglia) mediated by antipsychotic drugs and CLZ seems to emerge. Again, neuroinflammation cannot be considered the starting point. Therefore, we could speculate that sustained and dysfunctional inflammation represents a possible result of an original phenomenon of maladaptive coupling. Thus, the greater effectiveness of CLZ may be better explained by its ability to affect such a fundamental condition in several ways, including its ability to additionally foster an anti-inflammatory response.

Moreover, CLZ is rarely considered as a first-choice treatment for psychosis in human also because of its peculiar tolerability profile, including cardiovascular [80] and metabolic [81] side-effects, and, above all, the risk of CLZ-induced agranulocytosis/granulocytopenia (CIAG). CIAG manifests typically in first 18 weeks of treatment and may represent two possible phenotypes: CLZ-induced neutropenia (CIN) (absolute neutrophil count (ANC) ≤ 1500/μl) and CLZ-induced agranulocytosis (CIA) (ANC ≤ 500/μl) [82]. The cumulative risk of these blood dyscrasias is 3% and 0.8%, respectively [83]. Several studies recorded the incidence of agranulocytosis in patients treated with CLZ around 0.4% [84]. Interestingly, CLZ is not the only antipsychotic that could induce agranulocytosis. For example, some studies reported the same condition after olanzapine administration [85], although the incidence is much lower than that of CLZ [86]. Indeed, the mechanism of CIAG seems to be dose- independent and linked to an important genetic predisposition, with the involvement of HLA alleles [82, 87-90]. Despite the identification of possible involved alleles, however, the heritability of CIAG cannot be only explained and predicted, due to the polygenetic architecture of the condition.

One of the best- confirmed origins of CIAG seems to be related to CLZ metabolites [82]. CLZ metabolites are the pharmacologically active N-desmethylclozapine (NDMC), the inactive CLZ N-oxide (CNO), a reactive oxygen species, and the nitrenium ion. It is generally accepted that NDMC could cause neutropenia through different mechanisms associated with direct toxicity [91], rather than through the activation of immune system. Despite this evidence, the precise role of NDMC in the pathogenesis of CIAG is still unconfirmed. Nowadays, one of the most accredited theories on CIAG (either with CLZ and olanzapine) attributes the pathogenesis to the chemically reactive metabolite - nitrenium ion [92], resulting from the dehydrogenation of the piperazine ring. This chemical reaction could be mediated by NADPH oxidase/myeloperoxidase (MPO) system *via* neutrophil-generated hypochlorous acid. Of note, microglia elements, as mononuclear phagocytic cells, can express high levels of superoxide-producing NADPH oxidases (NOX) [93].

The pathogenesis of CIAG is not solely attributable to a single factor such as enzymatic activity; hence, numerous other mechanisms have been suggested, but the discussion of these possible mechanisms is not among the purposes of this paper. Curiously, CLZ induces early transient neutrophilia, paired with increases in serum granulocyte-colony stimulating factor (G-CSF) [94]. The phasic increments in G-CSF blood concentration are closely linked to CLZ administration and G-CSF is characterized by a short half-life. Thus, large spikes in G-CSF have been observed in different animal models at different time points, following CLZ administration [95].

Even in the absence of agranulocytosis, there is evidence suggesting an altered bone marrow function in the peripheral blood of CLZ-treated patients, including elevated numbers of CD34+ hematopoietic stem and progenitor cells, and decreased neutrophil nuclear lobe count [96, 97]. Apart from the idea of CLZ (and other antipsychotics) direct/indirect toxicity, some data from animal models (*i.e.,* rabbit) seem to indicate that CLZ stimulates the bone marrow to produce more neutrophils in a manner that is characteristic of endogenous G-CSF stimulation, thus directly or indirectly promoting differentiation and maturation of neutrophils in the bone marrow [95]. The link between CLZ and G-CSF is still not clarified. As known, G-CSF regulates both the proliferation and differentiation/activation of granulocytes, with associated peculiar molecular changes [98]. Of note, G-CSF stimulation promotes the expression of CD11b/ITGAM on neutrophils. Some authors have proposed that failure of the bone marrow to compensate for a shorter neutrophil half-life (altered neutrophil kinetic) could contribute to agranulocytosis [99]. GM-CSF, M-CSF and G-CSF also affect microglia in a complex way [100-102] and G-CSF seems capable of acting differentially on specific microglial phenotypes, increasing protective cytokine IL-4 production and inhibiting the productions of NO and other inflammatory mediators (IFN-γ, TNF-α, IL-1β, IL-17, and chemokine MCP-1) and the expression of MHC-II after LPS stimulation [101].

Although the evidence is eclectic and multifaced, we hypothesize that the superior effectiveness of CLZ in comparison to other antipsychotic medications might be related, at least in a subset of patients with SCZ, to the diversity of potential drug-induced immunomodulatory effects on dis-incorporated and aberrant microglia that we try to summarize:

- A more direct inhibitory effect on the acquisition of a dysfunctional pro-inflammatory phenotype.
- An indirect inhibitory action oriented at the purpose, potentially mediated by a more favourable ratio of DRs expressed by microglia. Such an action could crucially promote a renovation of more functional forms of coupling between microglia and CNS environment.
- A further possible killing effect - mediated by reactive metabolites, *e.g.,* nitrenium ion - perhaps more effective on certain aberrant/immature cellular phenotypes.
- A potential action favouring cell maturation and differentiation, which could be expressed through the induced spikes of growth factors (*e.g.,* G-CSF). These last two hypotheses are in line with the abovementioned results proposed by Snijders *et al*. [14], concerning the ‘immaturity’ of microglia in SCZ.

All these drug-induced/related phenomena may be only partially shared by other antipsychotic drugs; thus it is worth emphasizing the potential significance of the tricyclic molecular configuration in facilitating these effects. Tricyclic compounds are extremely diffused in clinical practice and, although sometimes considered exclusively in relation to CNS pathologies, many of them are also used for different conditions (*e.g.,* anti-infective and anti-cancer agents, as well as cardiovascular or anti-inflammatory drugs) [103]. Indeed, olanzapine and quetiapine share tricyclic structural characteristics with CLZ, and these structural similarities might explain cross-reactivity phenomena as well as similar adverse reactions such as agranulocytosis, probably underpinned by common immunological effects. However, as tricyclic structure effects on the immune system are particularly complex and still largely unknown, more studies are required to understand the putative pathogenetic mechanisms [104]. Still, regarding tricyclic antidepressants, results from several studies showed that their mechanism of action is complex and probably only partially related to their influence on neurotransmitters at the synaptic level, since these compounds are also capable of interacting with the immune system [105, 106]. Hence, expressing a ‘privileged structure’, and being ‘able to provide high-affinity ligands for more than one type of receptor’ [107], these drugs could still represent a fundamental means of treating novel emerging neurobiological conditions.

Through a deeper knowledge of its immunomodulatory action, CLZ, albeit its peculiar tolerability profile, may not be restricted to treatment-resistant SCZ. Indeed, a better understanding of the interactions between this psychotropic drug and the immune system could help clinicians to avoid CLZ use in the presence of some specific risk factors for dangerous reactions (CIAG) and could also be of significance in clarifying SCZ etiopathogenesis and, consequently, shedding light on possible novel therapies which may involve an immunomodulant activity.

## 3 OTHER RELEVANT PHARMACOLOGICAL ACTIONS

In analogy with other psychiatric conditions, such as the depressive spectrum and suicidal behaviour, the efficacy of augmentation strategies with anti-inflammatory/immune-modulating medications on SCZ symptomatology has been the main target of some recent meta-analyses [108, 109]. However, meta-analytical results are often quite difficult to interpret because of both the complexity of SCZ and other factors poorly considered by researchers (*e.g.,* baseline immune status, validity of diagnosis, disease phases stratification). We focused on some specific compounds better considered in the most recent meta-analytical reviews, even if other immunomodulant drugs tested during last years have shown a promising effect on SSDs symptoms (including fatty acids, melatonin, N-acetylcisteine, pioglitazone, piracetam and *Withania somnifera*).

### 3.1 Neuroactive Steroid and Schizophrenia

A subgroup of steroid hormones seems to influence many CNS structural and functional aspects [110-112], crossing BBB or being synthetized within the NS [113, 114]. The term ‘neurosteroids’ or ‘neuroactive steroids’ is collectively used for steroids deriving either from the circulation or synthesized *de novo in situ*, given the evidence for an indistinguishable effect into the CNS [110-117]. 17β-estradiol, dehydroepiandrosterone (DHEA), progesterone and allopregnanolone are the most studied and more abundant steroids in CNS, with a cell type-, region-, developmental stage- and gender-specific overall expression [110], mediated by astrocytes, oligodendrocytes, microglia and neurons [118-121].

Microglia express all three estrogenic receptors, ERα, ERβ and GPR30 [121-128], as well as PGMRC1/2 and mPRα for progesterone [129-131] and GABA-A receptor [132], which is the binding target of allopregnanolone [133, 134]. DHEA is a precursor of androgens and estrogens, thus its metabolites might mediate its effects in a very complex manner. In a recent narrative review, Yilmaz and colleagues [112] described how neurosteroids, 17β-estradiol, DHEA and allopregnanolone, might regulate neurodegeneration and neuroinflammation in many neuropathological conditions such as MS, Alzheimer’s and Parkinson’s diseases, and traumatic brain injuries (TBI), supporting neuronal survival either through a direct effect on neurons or by halting inflammatory responses of microglia and astrocyte, enhancing the expression of specific membrane/transmembrane receptors, inhibiting the expression of pro-inflammatory genes, and down-regulating inflammasome activation [112].

The immunomodulatory effect of steroid compounds, including novel DHEA analogues, on neuroinflammation and more specifically on microglia, has been deeply studied in recent years through *in vitro* models, animal models and knockout animal models [112, 122, 125, 135-144]. The novel synthetic DHEA analogues, recently renamed as “microneurotrophins” are of particular interest due to their small molecular size and selective functions, also expressed through neural growth factor (NGF) receptors [145], due to their small molecular size and their selective functions, also expressed through neural growth factor (NGF) receptors. Across different experimental models, 17β-estradiol, progesterone, allopregnanolone and DHEA were shown to reduce microglial inflammation.

Regarding the models of neurodegenerative diseases, the overall effect seems to be different for each steroid hormone [112]. Furthermore, eleven studies [146-156] investigated the effect of estrogens (4 = ethinyl-estradiol [147, 150-152]; 2 = conjugated estrogen [148, 154]; 5 = raloxifene, a selective estrogen receptor modulator [148, 152, 154-156]) as add-on therapy in SCZ, nine of these include only female subjects and only two males [148, 156]. The metanalysis [108] conducted on ten of the abovementioned works (*i.e*., after the exclusion of an outlier study [147]) provided a medium and significant mean weighted effect size (ES) of 0.57 (CI 0.25-0.90; *p* = 0.001; I2 = 74%) *vs* placebo for a relatively short duration of treatment (starting at 4 weeks). The ES remained significant and medium also after a restriction to female studies only (ES: 0.52; CI 0.18-0.87; *p* = 0.003; I2 = 72%). A meta-regression analysis showed that such a beneficial effect was primarily associated good- quality studies [108].

Apart from the aforementioned meta-analytical data, most of the clinical studies have been unable to demonstrate a definitive advantageous effect of systemically administrated steroid hormones in the progression of principal neurodegenerative diseases, including SSDs. For example, in a recent double-blind, placebo-controlled clinical trial [157], patients affected by SCZ, schizoaffective disorder or psychosis not otherwise specified (NOS) were randomized 1:1 to either prednisolone or placebo, in add-on therapy to their regular antipsychotic medication: after 6 weeks of treatment, symptom severity, measured through the Positive and Negative Syndrome Scale (PANSS) total score, decreased significantly in both the prednisone and placebo treatment arm (*p* < 0.001) but the degree of improvement was not significantly different between the two treatments arms (*p* = 0.96).

However, this lack of clinically significant results in human clinical trials could be attributed to many methodological and theoretical confounding factors, such as: a) the advanced disease stages of enrolled patients and the relatively low steroids administrated doses concentrations, compared to animal models, b) the heterogeneity of experimental samples, and c) the different mechanisms driving human and animal diseases, as well as the different physiological characterization between humans and animals. Finally, some steroids could not be particularly safe either for long-term (> 1-2 months) [158] or high dose short term treatments [159], giving a potential risk for cardiovascular adverse events, especially in SCZ subjects, that already express a higher cardio-metabolic risk [160, 161].

### 3.2 Other Proper Immunomodulant Drugs and Schizophrenia

The efficacy of nonsteroidal anti-inflammatory drugs (NSAIDs) as augmentation therapy in SCZ subjects was recently investigated [162]. Cyclooxygenase-2 (COX-2), the target enzyme subtype of some NSAIDs, was expressed in rat astrocytes and microglia as well as in experimental neuropathological conditions [163, 164], while its complex and differential expression in human NS is currently an ongoing line of research [164]. In a 6-week prospective double-blind randomized controlled trial (RCT), the add-on beneficial effect of celecoxib (COX-2 inhibitor) was proved in patients treated with risperidone during a relapse episode in SCZ [165], with an interesting positive effect on cognition [166]. These results were confirmed by a following study conducted in patients with first SCZ manifestations treated with amisulpride [167].

Regarding disease duration, a recent meta-analysis [168] highlighted a significant positive effect of NSAIDs in FEP but not in chronic stages of SCZ [169], while, as for dosage, high-doses (1 g/daily - poor BBB permeability [170]) of acetylsalicylic acid (ASA) add-on therapy seemed to positive influence total SCZ symptoms severity with an estimated ES of 0.3 (*vs* placebo) [108]. Therefore, specific clinical conditions, such as acute disease phase and shorter disease duration, seem to be related to NSAIDs efficacy. Of note, COX-2 inhibitors act on a very specific biochemical point within complex immunological pathways. This latter observation, and the proposed non-primary role of inflammation alone in SCZ pathogenesis could be related to the limited favourable effect identified in the previously mentioned studies.

The potential neuroprotective effect promoted by the second-generation tetracycline antibiotic minocycline against many neuropathological conditions has been investigated in multiple animal models [171-176]. This compound seems to cross the BBB [177] and to partially inhibit both ‘resting’ microglia proliferation [178] and M1 polarization [179]. Furthermore, minocycline inhibits glutamate-induced microglial ‘activation’ *via* the p38 mitogen-activated protein kinase pathway, providing neuroprotection against glutamate-induced excitotoxicity [180], and promotes dose-dependent positive effects on cognition in methamphetamine (METH) treated mice (animal model of METH psychosis and SCZ) [181]. Interestingly, METH-induced cognitive impairment in animal models was also found to be reversible through CLZ administration differently from haloperidol [182]. Similar results on cognition emerged from works conducted both on MK801 and on phencyclidine-model of SCZ [183, 184]. In contrast, other experimental studies showed a potential deleterious action at early postnatal stages in murine models [185-188].

Multidimensional positive effects (*e.g*., FEP, cognition, negative symptoms) of minocycline-based add-on strategies in treating SCZ, were reported by several studies and confirmed by two recent meta-analyses, together with an acceptable safety and tolerability profile [112, 189-197]. In contrast, although there are significant methodological issues, findings from a recent double-blind RCT [198] testing minocycline as adjunctive treatment for negative symptoms in FEP of schizophreniform disorder, or SCZ within 5 years of illness onset, seem to question minocycline’s clinical efficacy [199, 200].

Finally, in 2020, Jeppesen and colleagues conducted the largest and most comprehensive meta-analysis on anti-inflammatory add-on treatment to antipsychotics for SSDs, including seventy RCTs studies, that investigated either primarily anti-inflammatory drugs (*i.e*., NSAIDs, minocycline and monoclonal antibodies), or drugs with potential anti-inflammatory properties (*i.e*., neurosteroids, N-acetyl cysteine - NAC, estrogens, fatty acids, statins, and glitazones) [201]. Anti-inflammatory add-on treatment, compared to antipsychotics plus placebo, was associated with a PANSS scale mean difference (MD) improvement of -4.57 (95%CI = -5.93 to -3.20) points (standardized mean difference -SMD- ES of -0.29, 95%CI = -0.40 to -0.19). Significant effects were also found on negative symptoms (MD = -1.29), positive symptoms (MD = -0.53), general psychopathology (MD = -1.50) and working memory (SMD = 0.21). Primarily anti-inflammatory drugs (MD = 4.00; 95%CI = -7.19 to -0.80) were not superior (*p* = 0.69) to potential anti-inflammatory drugs (*e.g*., NAC, statins) (MD = 4.71; 95%CI = -6.26 to -3.17), even if metaregression found that smaller studies showed significantly larger effect sizes than the larger studies (*p* = 0.0085). Interestingly, the beneficial effect on symptoms severity and cognition of some non-classical anti-inflammatory compounds (*e.g*., NAC, statins) also emerged from the above-mentioned meta-analysis [108, 109], especially in early phases of SCZ. From our perspective, this last convergence of results supports the idea that neuroinflammation is not the primary pathogenetic factor in SSDs. Rather, it may be a dysfunctional manifestation of an underlying issue, on which different classes of drugs could act, promoting the reconstitution of a more integrated state of microglia into CNS environment.

## CONCLUSION

In line with recent experimental evidence focusing on the role of neuroinflammation and microglia in SCZ and SSDs pathogenesis, an immunomodulant approach represents an emergent and promising augmentation strategy in treating these conditions.

In this narrative review, we discuss the potential benefits of combining classical psychotropic drugs with compounds that have a direct effect on the immune system. Specifically, we highlight the importance of considering the synergic immunomodulant effect, potentially inducible with combinations of classical psychotropic drugs (*e.g.,* second- generation antipsychotics, with a specific focus on CLZ, or tricyclic antidepressants) and the other above-mentioned compounds acting more directly on the immune system. In particular, the greater positive effect of CLZ in SSDs may be due to its ability to act on aberrant microglia.

However, it should be noted that inflammatory mechanisms might only be involved in certain subgroups of SSDs patients. Indeed, some studies have suggested that immunomodulatory treatments are more efficacious in subjects suffering from SCZ with elevated inflammatory markers [168, 202], while others have reported a decrease in pro-inflammatory cytokines related to symptoms’ improvement [203].

In addition, also some immunomodulant drugs seem to be more clearly effective (*e.g*., aspirin, estrogens, minocycline) [108], with an interesting beneficial action potentially exerted on negative symptoms and cognition.

At the same time, non-classical immunomodulatory compounds may also be beneficial in treating these disorders, as they can target deeper pathological processes, thus suggesting the importance of considering the dysfunctional inflammation as a secondary manifestation of more profound pathological phenomena (*e.g.,* microglia dis-incorporation). Further studies are needed to better define the efficacy, safety, and actual effectiveness of these drugs, within different treatment strategies.

Finally, recent works reported that the efficacy of most of the drugs explored in this review seems to be greater on symptoms’ severity in FEP or early-phase SCZ [108]. Therefore, future research should consider disease progression and clinical staging to evaluate the potential beneficial effects of immunomodulatory drugs regarding more specific phases of these psychopathological disorders.

## LIST OF ABBREVIATIONS

BBB: Blood-brain Barrier
BD: Bipolar Disorder
CNS: Central Nervous System
DRs: Dopamine Receptors
FEP: First Episode of Psychosis
MHC: Major Histocompatibility Complex
MIA: Maternal Immune Activation
MS: Multiple Sclerosis
NS: Nervous System
NSAIDs: Nonsteroidal Anti-inflammatory Drugs
PANSS: Positive and Negative Syndrome Scale
SCZ: Schizophrenia
SSDs: Schizophrenia Spectrum Disorders
TBI: Traumatic Brain Injuries
